# CRISPR/Cas9-mediated genome editing directed by a 5S rRNA–tRNA^Gly^ hybrid promoter in the thermophilic filamentous fungus *Humicola insolens*

**DOI:** 10.1186/s13068-021-02057-y

**Published:** 2021-10-23

**Authors:** Chao Fan, Wei Zhang, Xiaoyun Su, Wangli Ji, Huiying Luo, Yuhong Zhang, Bo Liu, Bin Yao, Huoqing Huang, Xinxin Xu

**Affiliations:** 1 Biotechnology Research Institute, Chinese Academy of Agricultural Sciences, No. 12 South Zhongguancun St., Haidian District, Beijing, 100081 China; 2 Institute of Animal Sciences, Chinese Academy of Agricultural Sciences, No. 2 West Yuanmingyuan Road, Haidian District, Beijing, 100193 China

**Keywords:** *Humicola insolens*, CRISPR/Cas9, 5S rRNA, tRNA^Gly^, Cellulase

## Abstract

**Background:**

*Humicola insolens* is a filamentous fungus with high potential of producing neutral and heat- and alkali-resistant cellulase. However, the genetic engineering tools, particularly the genome-editing tool, are scarce, hindering the study of cellulase expression regulation in this organism.

**Results:**

Herein, a CRISPR/Cas9 genome-editing system was established in *H. insolens* based on a hybrid 5S rRNA–tRNA^Gly^ promoter. This system is superior to the HDV (hepatitis delta virus) system in genome editing, allowing highly efficient single gene destruction in *H. insolens* with rates of deletion up to 84.1% (37/44). With this system, a putative pigment synthesis gene *pks* and the transcription factor *xyr1* gene were disrupted with high efficiency. Moreover, the extracellular protein concentration and cellulase activity largely decreased when *xyr1* was deleted, demonstrating for the first time that Xyr1 plays an important role in cellulase expression regulation.

**Conclusions:**

The established CRISPR/Cas9 system is a powerful genetic operation tool for *H. insolens*, which will accelerate studies on the regulation mechanism of cellulase expression and engineering of *H. insolens* for higher cellulase production.

**Supplementary Information:**

The online version contains supplementary material available at 10.1186/s13068-021-02057-y.

## Background

Lignocellulose is one of the most abundant renewable biomass resources on earth, which contains cellulose, hemicellulose, and lignin as its major components. Cellulase and hemicellulase degrade the two plant cell wall polysaccharides into simple sugars including mono sugars or oligosaccharides, which can be used by natural occurring or engineered brewer’s yeasts to produce ethanol and advanced biofuels. This leads to an eco-friendly solution to the current energy and environmental problems [1]. The thermophilic filamentous fungus *Humicola insolens* is thus regarded to be of high potential, because it has noticeable merits such as high growth temperature, fast growth rate, and excellent cellulase- and hemicellulase-producing ability [2]. Its cellulase system was similar to that of *Trichoderma reesei*. However, the straw degradation efficiency of the *H. insolens* cellulase was higher than that of *T. reesei*. In addition, the *H. insolens* cellulase has stable activity at high temperature [2, 3]. The high temperature-resistant β-glucosidase and xylanase expressed by *H. insolens* have been used in the wine industry for quality improvement, while its neutral cellulase has been used in the textile and washing industry [4]. By having excellent heat- and alkali-resistance, high cellulose degradation ability, and an optimal pH close to neutral, the cellulase expressed by *H. insolens* is a good complement to that from *T*. *reesei* [4–7]. However, the production level of *H. insolens* is low and cannot meet the need of biofuel industries. Previously, a T-DNA random insertional mutant library created by *Agrobacterium tumefaciens*-mediated transformation was established [8]. It was also discovered that mutation of the transcriptional regulator CreA did not greatly improve the ability of *H. insolens* to produce cellulase [9]. One main reason for the inefficiency of strain engineering is that there is no well-established genome-editing system, limiting the study of the regulating mechanisms of cellulase expression in *H. insolens*. There is an urgent need for a new technology to solve this problem.

With the advantages of high efficiency, versatility and ease of operation, the CRISPR/Cas9 technology is now widely used in functional genomics studies of filamentous fungi [10–12]. The essence of this technology is that, a small guide RNA (sgRNA) is designed to target and direct the Cas9 nuclease to bind and cleave a specific site in the chromosome [13]. sgRNA recognizes and complexes with the DNA in the targeting site. The complex is inserted into the gap between the nuclease recognition and cutting sites of Cas9, which will activate the cleavage activity of Cas9 and lead to cutting of the target site and forming a double-stranded break (DSB) [14, 15]. DSB is repaired by either the non-homologous end joining (NHEJ) or homology-directed repair (HDR) mechanisms [16].

Herein, the CRISPR/Cas9-based genome-editing technique was established for the first time in *H. insolens*. The technique contains a codon-optimized Cas9 nuclease-expressing cassette directed by a *tef1* promoter, a sgRNA-expressing cassette with a prevailing tRNA^Gly^ element directed by a 5S rRNA promoter, and a donor DNA fragment with 600-bp homologous arms. Using this system, we greatly improved the genome editing efficiency in *H. insolens* and successfully disrupted the *pks* pigment synthesis gene and *xyr1* transcription factor gene, with a maximum efficiency of 84.1% (37/44) and 78.3% (18/23), respectively. This method displayed great potential for genome editing in *H. insolens* and laid a foundation for functional genomics as well as construction of engineered strains with improved ability to produce cellulase.

## Results and discussion

### Construction of a CRISPR/Cas9 system for genome editing in *H. insolens*

**Fig. 1:**
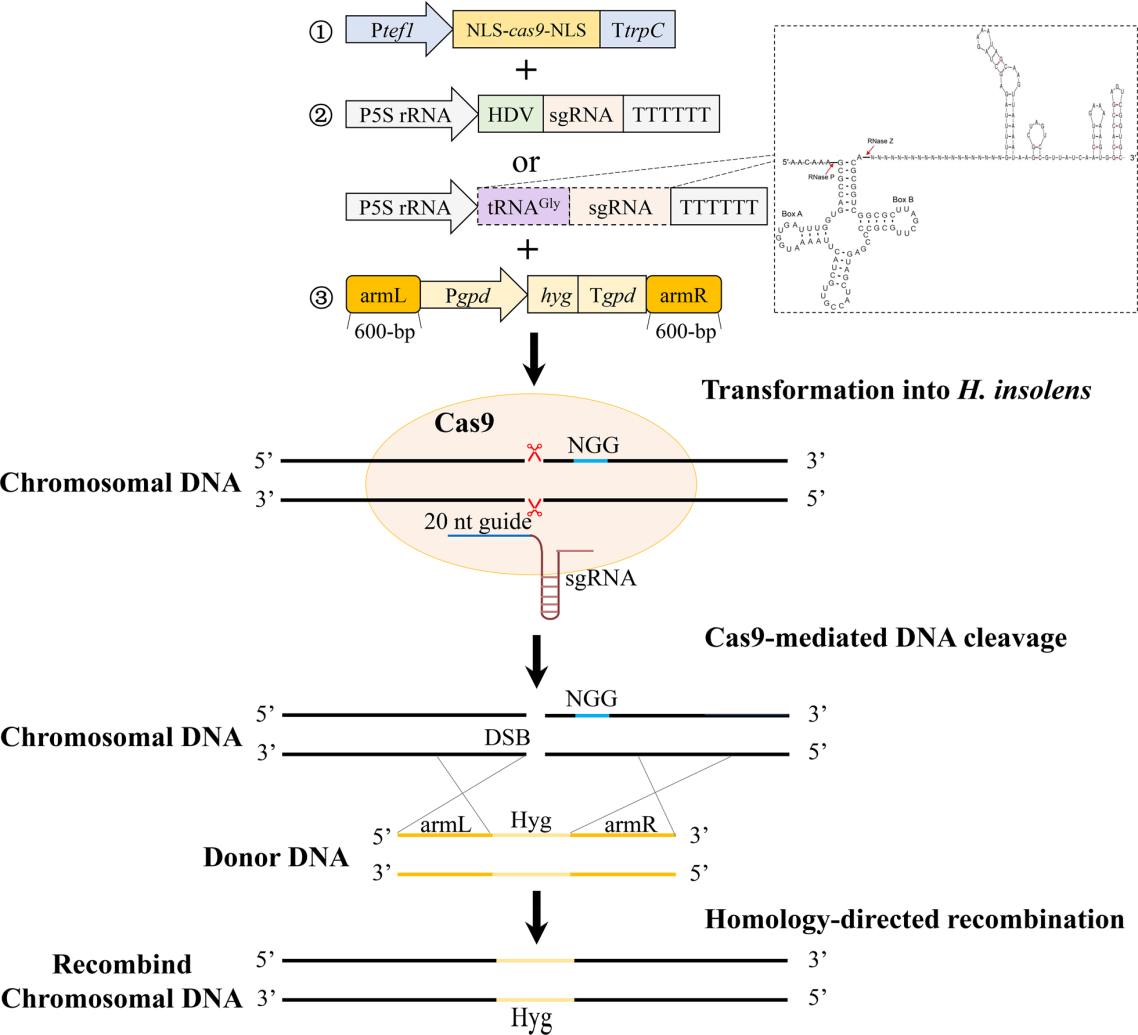
Schematic representation of the CRISPR/Cas9 genome editing systems in *H. insolens.* ① Cas9 expression cassette. P*tef1*: *tef1* promoter from *M. thermophila*. ② sgRNA expression cassette. P5S rRNA (light gray): the *A. niger* 5S rRNA gene with its 338-bp upstream promoter. HDV (light green): the hepatitis delta virus ribozyme; tRNA^Gly^ (purple): the transfer RNA for glycine from *H. insolens*. The dotted box frames up the structure of tRNA^Gly^–sgRNA; the red arrows indicate excision sites by RNase P and RNase Z. ③ Donor DNA. armL (bright brown): the upstream homologous arm; armR (bright brown): downstream homologous arm; NGG (blue): the PAM sequence; DSB: double-strand break; Hyg (light brown): hygromycin B resistance gene expression cassette

The CRISPR/Cas9 system used in *H. insolens* included a Cas9-expressing cassette, a sgRNA expression cassette, and a donor DNA fragment (Fig. 1). Abundant Cas9 protein and sgRNA are well-known to be critical to successful genome editing. Since expression of Cas9 depends heavily on the promoter, the strong and constitutive promoter P*tef1* has been successfully applied in Aspergilli (the *Aspergillus nidulans* P*tef1*) and *M. thermophile* (the *Myceliophthora thermophila* P*tef1*) [11, 17]. Therefore, the Cas9-expressing cassette containing the *tef1* promoter, *cas9* gene from *Streptococcus pyogenes* with two nuclear localization signals (NLS), and the *trpC* terminator was synthesized according to the sequence of Cas9-expressing cassette used in *M. thermophila* [17]. It was used herein to provide stable expression of Cas9 and proved to have a good effect on gene editing (Fig. 1; Table 1).

**Table 1:** The genome editing efficiency of CRISPR/Cas9 system for *H. insolens* in this study

Host strain	Target gene	DNA fragments used for transformation		Number of target gene disruption transformants	Number of analyzed transformants	Number of all transformants	Gene editing efficiency (%)
Y1	*pks*	Cas9 + sgRNA	Cas9 + 5SsgRNA-*pks1*	14	345	557	4.1
			Cas9 + tRNAsgRNA-*pks1*	35	375	594	9.3
			Cas9 + 5SsgRNA-*pks1*-HDV	26	371	590	7.0
			Cas9 + 5SHDVsgRNA-*pks1*	79	336	527	23.5
			Cas9 + 5StRNAsgRNA-*pks1*	186	322	534	57.8
			Cas9 + 5SHDVsgRNA-*pks2*	71	317	536	22.4
			Cas9 + 5StRNAsgRNA-*pks2*	194	328	549	59.1
			Cas9 + 5SHDVsgRNA-*pks3*	60	281	492	21.4
			Cas9 + 5StRNAsgRNA-*pks3*	208	369	611	56.4
		Donor DNA + sgRNA	Donor-*pks* + 5SsgRNA-*pks1*-HDV	11	363	586	3.0
			Donor-*pks* + 5SHDVsgRNA-*pks1*	11	351	556	3.1
			Donor-*pks* + 5StRNAsgRNA-*pks1*	17	394	618	4.3
		Donor DNA + Cas9	Donor-*pks* + Cas9	18	383	617	4.7
		Cas9 + donor DNA + sgRNA	Cas9 + donor-*pks* + 5SHDVsgRNA-*pks1*	91	342	562	26.6
			Cas9 + donor-*pks* + 5StRNAsgRNA-*pks1*	204	363	593	56.2
		Donor DNA	Donor-*pks*	25	400	625	6.3
Y1/*Δku70*	*pks*	Donor DNA	Donor-*pks*	8	73	143	11.0
		Cas9 + donor DNA + sgRNA	Cas9 + donor-*pks* + 5SHDVsgRNA-*pks1*	10	39	92	25.6
			Cas9 + donor-*pks* + 5StRNAsgRNA-*pks1*	37	44	85	84.1
	*xyr1*		Cas9 + donor-*xyr1* + 5SHDVsgRNA-*xyr1*	6	23	76	26.1
			Cas9 + donor-*xyr1* + 5StRNAsgRNA-*xyr1*	18	23	81	78.3
	*pks* + *xyr1*		Cas9 + donor-*pks* + donor-*xyr1* + 5StRNA sgRNA-*pks1* + 5StRNAsgRNA-*xyr1*	4	23	38	17.4

For sgRNA expression in other fungi such as *T. reesei*, *Neurospora crassa*, *Aspergillus fumigatus*, *Penicillium chrysogenum*, and *Pyricularia oryzae*, typically an RNA polymerase III type U6 promoter was used to drive its transcription [12, 18–21]. However, it was sometimes difficult to identify a U6 promoter in many species and the use of a heterologous U6 promoter may otherwise reduce the efficiency of gene editing [22, 23]. In addition to the U6 promoter, there are also other types of promoters successfully used to initiate sgRNA transcription. In *Aspergillus niger* and *Fusarium fujikuroi*, a highly conserved 5S rRNA promoter was found to drive sgRNA transcription with higher efficiency than that using the U6 promoter [24, 25]. Furthermore, high gene editing efficiency can be obtained by using 5S rRNA along with its upstream 338-bp sequence as the promoter in *A. niger* [25]. Four tRNA promoters were more efficient than the U6 promoter in *Ustilaginoidea virens* [26]. A small RNA, i.e., tRNA^Gly^, was regarded to be able to enhance transcription of the sgRNA as a potential enhancer of Pol III and also ensure precise release of sgRNA spacer-scaffold structure from the sgRNA expression cassette [27, 28]. In addition to change of the promoter, in recent years, ribozymes (an RNA-based nuclease) were included in the sgRNA expression cassette, expanding the use of other types of promoters. In Aspergilli, an HDV ribozyme was fused in the sgRNA expression cassette to liberate more sgRNA [11, 25]. Taken together, both the HDV ribozyme and the tRNA^Gly^ element were used in sgRNA expression cassette to improve the genome editing efficiency in different filamentous fungi. Since the U6 promoter of *H. insolens* was not identified at present, two strategies employing either the HDV ribozyme or tRNA^Gly^ element were used to improve sgRNA expression directed by the 5S rRNA (-338) promoter in this study (Fig. 1).

For the donor fragments, the length of homologous arms has a high impact on the efficiency of genome editing. It was reported that, when the homologous arms for *lea1* were 600-bp (or more) in *T. reesei*, the genome editing efficiency reached 100% [10]. Therefore, we designed homologous arms with 600-bp and fused them with the marker gene-expressing cassette P*gpd*-*hyg*-T*gpd* (Fig. 1).

### Selection of target genes for genome editing

The putative *pks* and *xyr1* genes were predicted from the genome of *H. insolens* and selected as targets of genome editing in *H. insolens* using the CRISPR/Cas9 system. The 6694-bp *pks* gene is predicted to encode a putative polyketide synthase (Pks) containing 2167 amino acids, which is necessary for the black pigment melanin biosynthesis [29]. The Pks from *H. insolens* is homologous to *Pestalotiopsis fici* PfmaE [30], *A. nidulans* AnWA [31], *A. niger* AnAlbA [32], and *A. fumigatus* AfAlb1 [33] with amino acid sequence identity of 61.9%, 37.6%, 39.4%, and 34.8%, respectively (Additional file 1: Figure S1). The 3403-bp *xyr1* of *H. insolens* encodes a transcription factor belonging to the MHR superfamily [34]. The predicted Xyr1 protein contains 969 amino acids typified by a Zn_2_Cys_6_ zinc finger motif [35]. The *H. insolens* Xyr1 has 78.7%, 74.2%, 55.6% and 48.4% amino acid sequence identity, respectively, to *M. thermophila* MtXyr1 [36], *N. crassa* NcXyr1 [37], *T. reesei* TrXyr1 [38], and *A. nidulans* AnXyr1 [39] (Additional file 1: Figure S2).

### 5StRNA^Gly^–sgRNA is superior to 5SHDV–sgRNA in genome editing

**Fig. 2:**
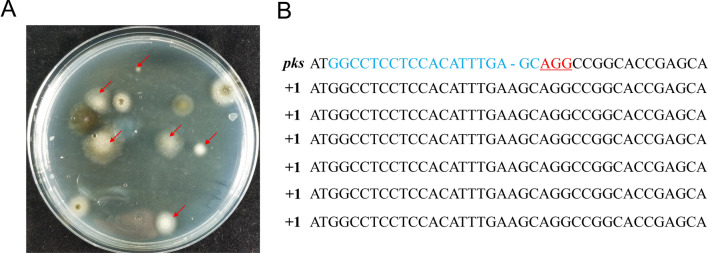
Mutations into the *pks* gene in *H. insolens* as introduced by CRISPR/Cas9-mediated genome editing. **A** Disruption of *pks* was correlated to an albino conidia phenotype in *H. insolens*. The *pks* gene disrupted strains displayed pigment-less conidia and resultant white colonies as indicated by red arrows. **B** Sequence alignment of the *pks* gene in WT and the *pks*-edited mutants. The 20-nt protospacer sequence of *pks* in wild-type strain is represented by blue letters and PAM sequence is labeled by red letters with underline

Similar to *A. niger*, the *pks*-destroyed mutants of *H. insolens* produced albino conidia (Fig. 2A). When the wild-type Y1 strain was used, the 5StRNA^Gly^–sgRNA (where 5StRNA^Gly^ is an abbreviation for the 5SrRNA–tRNA^Gly^ construct) expression disrupted 57.8% (186/322, *pks1*) of the *pks* gene in transformants without addition of a donor DNA, while the 5SHDV–sgRNA (where 5SHDV is an abbreviation for the 5SrRNA-HDV construct) system had a disruption efficiency of only 23.5% (79/336, *pks1*) (Table 1). This trend was stable for other sgRNAs, *pks*2 and *pks*3, two other target sites of *pks* gene (Table 1). The 5StRNA^Gly^–sgRNA system had an efficiency of 59.1% (194/328) for *pks2* and 56.4% (208/369) for *pks3*, while the efficiency of 5SHDV–sgRNA system were only 22.4% (71/317, *pks2*) and 21.4% (60/281, *pks3*). Six *pks* gene mutations were verified by sequencing. These mutants displayed insertion mutations upstream of the PAM site with a single-nucleotide A (Fig. 2B). The efficiency of genome editing did not change much (56.2% (204/363) for 5StRNA^Gly^–sgRNA and 26.6% (91/342) for 5SHDV–sgRNA) when the donor DNA was added.

In the wild-type Y1 strain, the numbers of transformation did not change significantly due to the addition of Cas9 expression cassette (617 transformants for donor DNA/Cas9 cotransformation versus 625 transformants for donor DNA only). Additionally, the controls without donor DNA using Cas9 expression cassette and 5SsgRNA–HDV (abbreviation for the 5SrRNA–sgRNA-HDV construct), 5SsgRNA (abbreviation for the 5SrRNA–sgRNA construct), tRNA^Gly^–sgRNA systems all displayed low disruption efficiency for 7.0% (26/371), 4.1% (14/345) and 9.3% (35/375), respectively (Table 1). Apparently, the efficiency of genome editing using the 5StRNA^Gly^–sgRNA system almost doubled that of 5SHDV–sgRNA (Table 1). This is worth noting since in *A. niger*, addition of the HDV ribozyme improves the genome editing efficiency [25]. Two reasons may account for the high efficiency of 5StRNA^Gly^–sgRNA system in *H. insolens*. For one thing, tRNA^Gly^ can recruit the PolIII complex due to the existence of internal promoter elements box A and B and drive transcription of sgRNA by serving as a promoter [40]. For another, the structure of pre-tRNA^Gly^ can be recognized by RNase P and RNase Z and the site between tRNA^Gly^ and sgRNA spacer is efficiently cleaved [41]. These lead to releasing of active guide RNA molecules and explain the higher cleavage activity than observed for HDV.

**Fig. 3:**
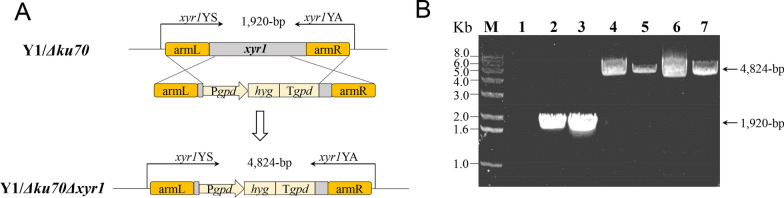
Verification of *xyr1* gene disruption in the Y1/*Δku70* strain. A Schematic diagram of *xyr1* disruption verification. *hyg*: hygromycin B resistance gene. B PCR verification of *xyr1* disruption using the primer pair *xyr1*YS/*xyr1*YA. M: DNA molecular mass marker; lane 1: negative control; lane 2: *H. insolens* Y1; lane 3: Y1/*Δku70*; lanes 4–7: four representative *xyr1* disrupted mutants

In cells, the broken DNA double strands are repaired through either the nonhomologous end joining (NHEJ) pathway or homology-directed repair (HDR) pathway [42]. The NHEJ pathway is rapid and does not require a template [43]. In fungi, the Ku proteins play a key role in the NHEJ pathway for DNA repair. When the DNA strands are broken, the Ku70/Ku80 heterodimer recognizes and binds to the broken ends and recruits kinase, ligase, and other associated proteins for DNA repairing [16, 44]. In filamentous fungi, it has been reported that disrupting the gene encoding Ku70 can significantly improve the efficiency of homologous recombination [21, 45–47]. Therefore, we used the Y1/*Δku70* strain to further test CRISPR/Cas9-mediated genome editing. In this strain, the gene-editing efficiency for *pks* using the 5StRNA^Gly^–sgRNA system increased from 56.2% (204/363, in the WT strain) to 84.1% (37/44), tripling that of 5SHDV–sgRNA (25.6%, 10/39, Table 1). Disruption of *xyr1* was all carried out by supplementing with the donor DNA (Fig. 3A) and this gene was disrupted at an efficiency of 78.1% (18/23) with 5StRNA^Gly^–sgRNA system but at 26.1% (6/23) with the 5SHDV–sgRNA system (Table 1). This again demonstrated that the using tRNA^Gly^ was more efficient in genome editing than HDV. Disruption of *xyr1* by homologous recombination was verified by diagnostic PCR: a 4824-bp specific fragment was amplified by PCR from the *xyr1* disruption mutant, while a 1920-bp fragment was amplified from the wild-type Y1 and Y1/*Δku70* strains (Fig. 3B).

When the Y1/*Δku70* strain was used as the host strain, the genome editing efficiency of the 5SHDV–sgRNA system was comparable for *pks*. In Y1/*Δku70* the knockout efficiency was 25.6% (10/39), while in Y1 the efficiency was 26.6% (91/342) (Table 1). With this strain, the increase of genome editing efficiency of the 5StRNA^Gly^–sgRNA system was obvious: 56.2% (204/363) in Y1 versus 84.1% (37/44) in Y1/*Δku70*. However, the efficiency still did not reach to nearly 100% as observed in *Magnaporthe grisea* and *A. nidulans* [48, 49]. Note that the genome editing efficiency of 11.0% (8/73) in Y1/*Δku70* did not change significantly as compared with 6.3% (25/400) in Y1 when only the donor DNA for *pks1* was used. There might be several reasons explaining the limited increase of genome editing efficiency in the *ku70* disrupted strain. First, the expression of *ku70* is so low that knockout of this gene does not have significant impact on the repair rate of NHEJ [50]. Second, the expression of HDR-related component genes is too low that the homology recombination efficiency cannot be improved even after the NHEJ pathway is repressed [51].

We also used the 5StRNA^Gly^–sgRNA system in the Y1/*Δku70* strain for multiplexing genome editing. In the trial to simultaneously destruct two genes, the successful rate of deleting *pks* and *xyr1* dropped to 17.4% (Table 1). These results revealed that this system could be used for editing the cellulase expression-related genes in *H. insolens*.

### Deletion of *xyr1* decreased cellulase production in *H. insolens*

Xyr1 is the main transcription activator of cellulase expression in mainly lignocellulose-degrading filamentous fungi [52, 53]. However, the regulatory role of Xyr1 on fungal growth and cellulase expression is not consistent in all filamentous fungi due to its phosphorylation degree [54, 55]. In *H. insolens,* the role of *xyr1* in cellulase expression regulation is not clear. Therefore, in this study, the *xyr1* mutant of *H. insolens* obtained by CRISPR/Cas9-mediated gene disruption was further studied for growth phenotype and cellulase production.

**Fig. 4:**
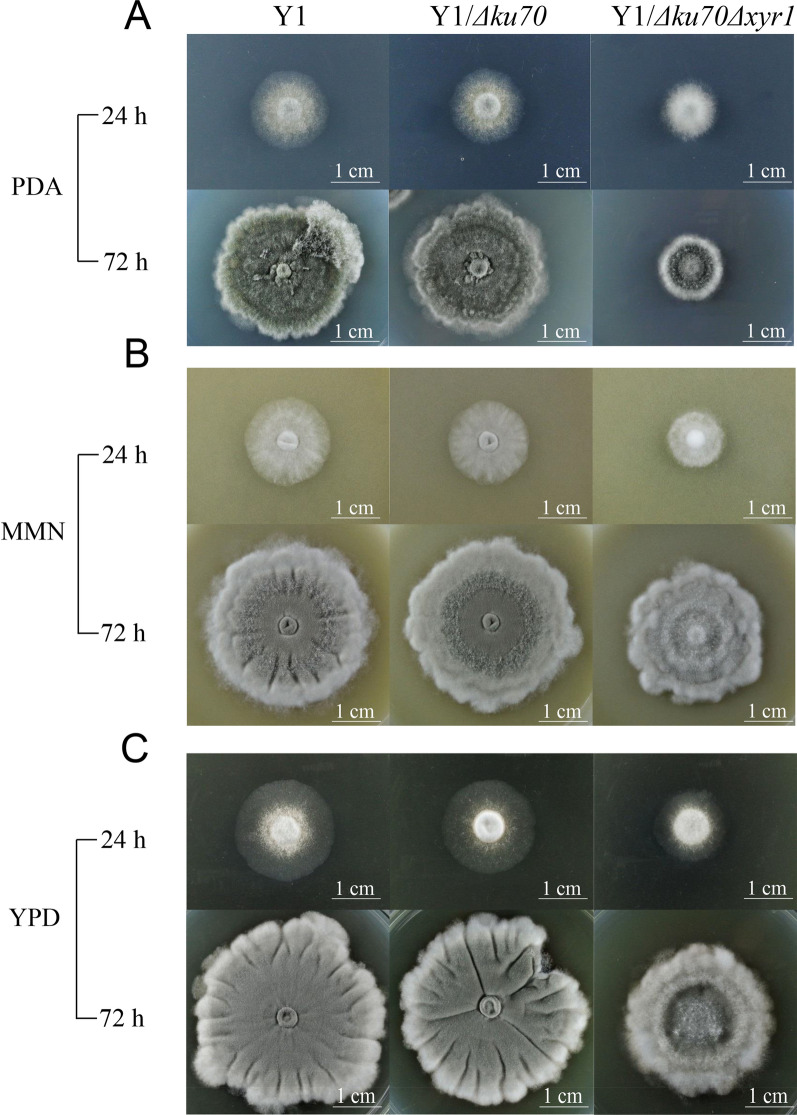
Characterization of the growth morphology of *xyr1* disruption mutants. Colony growth and sporulation of the wild-type Y1, Y1/*Δku70*, and Y1/*Δku70Δxyr1* strains cultured on PDA (**A**), MMN (**B**) and YPD (**C**) plates at 42 °C for 24 and 72 h, respectively

On culturing at 42 °C in PDA, MMN, and YPD solid plates, the nascent hyphae were dense and clustered in the center of the colonies. At 24 h, the colony of Y1/*Δku70Δxyr1* strain was significantly smaller than that of Y1 and Y1/*Δku70* strains. This was also true for colonies grown at 72 h on these plates (Fig. 4A–C). When cultured in PDA, the sporulation of Y1/*Δku70Δxyr1* was basically the same as those of wild-type and the Y1/*Δku70* strains (Fig. 4A). Although the sporulation of Y1/*Δku70Δxyr1* strain was slower, the length of its aerial hyphae appeared to increase (Fig. 4B) in the MMN medium. In YPD, Y1/*Δku70Δxyr1* showed a layered state of mycelial extension in addition to aerial hyphae (Fig. 4C). Deletion of *xyr1* and its homologous genes in other filamentous fungi such as *T. reesei*, *M. thermophila* and Aspergilli similarly altered the growth phenotypes depending on the carbon sources [55–57].

**Fig. 5:**
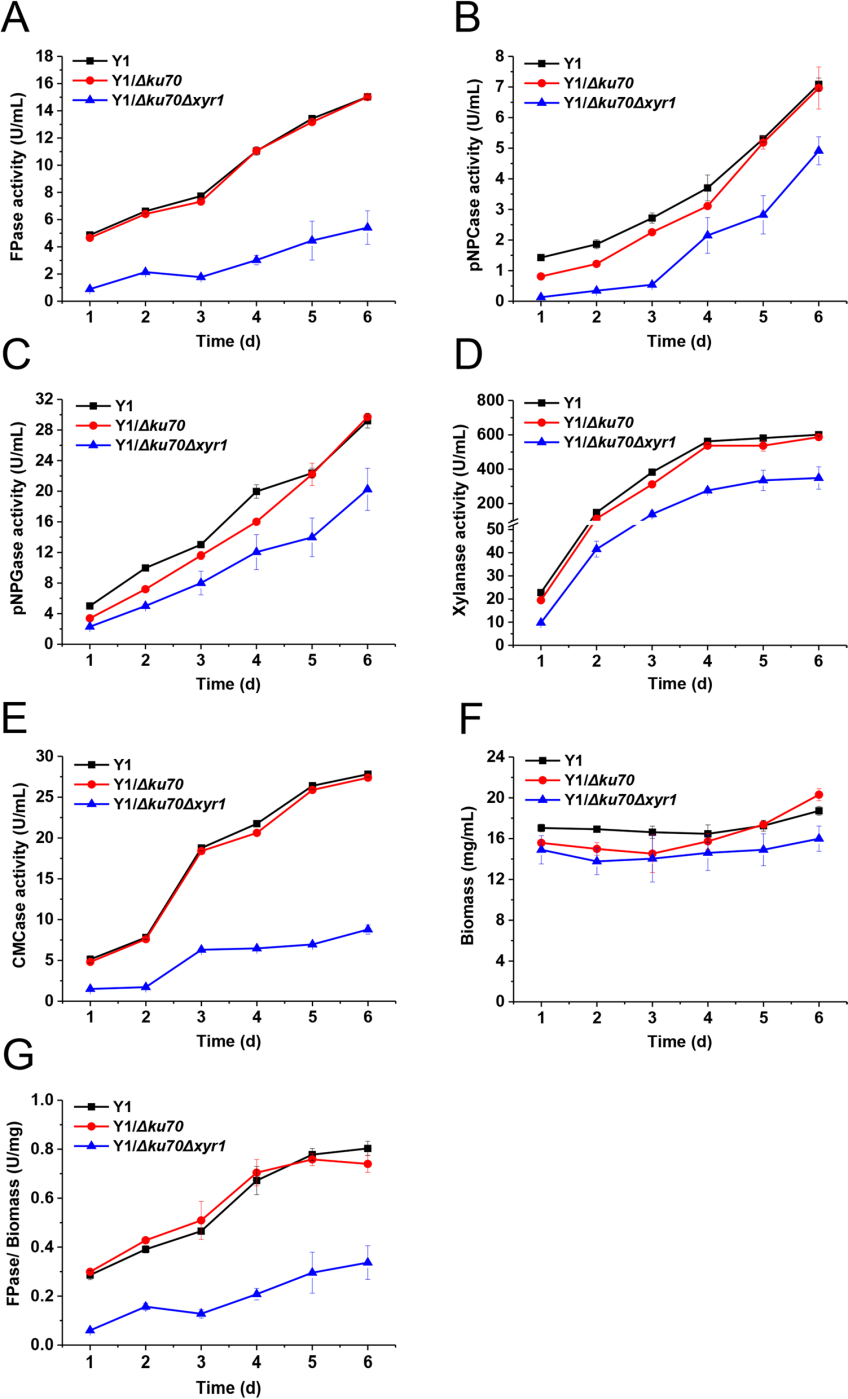
Assay of enzyme activities of *H. insolens*. The activities are FPase (**A**), CMCase (**B**), pNPCase (**C**), pNPGase (**D**), and xylanase (**E**). The biomass (**F**) and FPase/biomass (**G**) are also shown

In flask fermentation, the wild-type Y1 and Y1/*Δku70* strains produced almost the same amount of extracellular enzymes. However, both the cellulase and hemicellulase activities of the Y1/*Δku70Δxyr1* were lower than those of the wild type (Fig. 5). The FPase activity of Y1/*Δku70Δxyr1* reached the maximum on day 6 post-induction, 63.95% lower than that of WT (Fig. 5A). The enzymatic activities of endoglucanase, cellobiohydrolase, β-glucosidase, and xylanase of Y1/*Δku70Δxyr1* were also significantly decreased to as low as 32.10%, 70.55%, 68.16%, and 59.45%, respectively, of the original strain (Fig. 5A–E). The biomass of Y1/*Δku70Δxyr1* strain was slightly lower than that of Y1 throughout the culture. Y1/*Δku70* was similar to Y1/*Δku70Δxyr1* till day 4 post-induction, but then it grew to a level similar to that of the wild type (Fig. 5F). For Y1/*Δku70Δxyr1*, the highest FPase/biomass ratio was 0.34 U/mg, which was much lower than those of Y1 (0.80 U/mg) and Y1/*Δku70* (0.74 U/mg) (Fig. 5G).

**Fig. 6:**
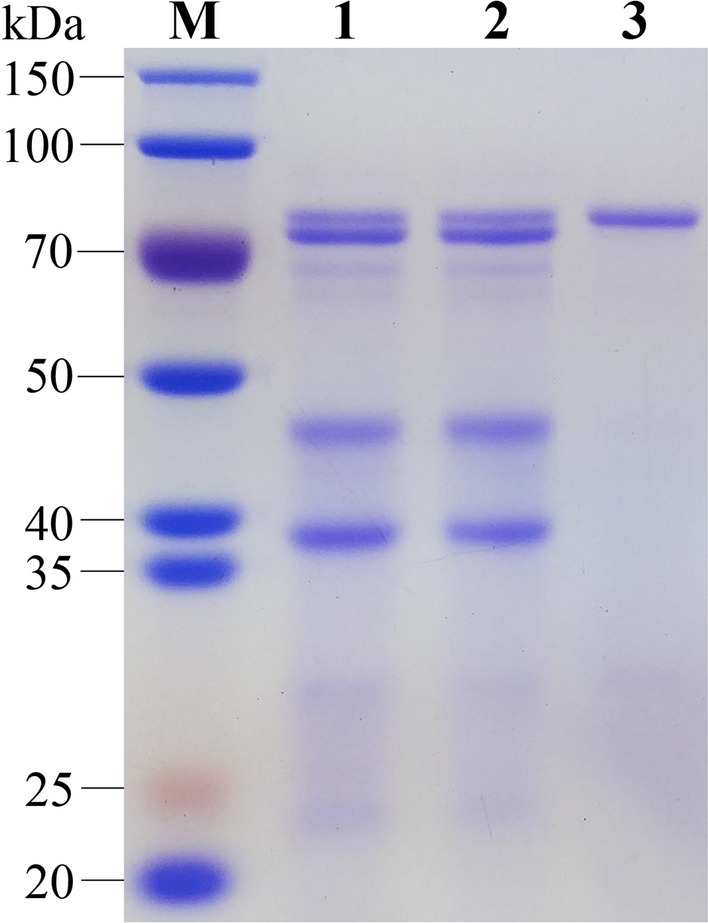
*xyr1* deletion changed the extracellular protein profile in *H. insolens*, as analyzed by SDS-PAGE. M: protein molecular mass marker; lane 1: the *H. insolens* Y1 strain; lane 2: Y1/*Δku70*; lane 3: Y1/*Δku70Δxyr1*

An SDS-PAGE analysis indicated that, compared with Y1 and Y1/*Δku70* strains, the major extracellular proteins with molecular masses lower than 70 kDa in Y1/*Δku70Δxyr1* almost completely disappeared (Fig. 6). Taken together, the decrease of enzyme activity in *H. insolens* Y1/*Δku70Δxyr1* was due to lowered ability to express the enzymes as well as the impaired biomass accumulation. These results were also consistent with the results of other filamentous fungi [56, 58], and for the first time, demonstrated that Xyr1 played a positive pleiotropic regulatory role in cellulase expression of *H. insolens*.

The widespread Xyr1 and its homologs can activate transcription of cellulase and/or hemicellulase genes in many lignocellulose-degrading filamentous fungi [59]. It is well-known that, after *xyr1* deletion, almost all cellulase and hemicellulase genes are unable to be induced in *T. reesei* [60]. However, deletion of *xlnR* (encoding a Xyr1 homolog) in *Fusarium graminearum* led to elevation of cellulase gene transcription [61]. The discrepancy in transcriptional regulation by Xyr1 is that the function of this transcription factor is mainly determined by its phosphorylation status, but also impacted by the interaction between Xyr1 and other transcription regulators [62–64].

## Conclusion

In this study, an efficient CRISPR/Cas9 genome editing platform for *H. insolens* has been successfully developed for the first time, employing the 5S rRNA promoter and tRNA^Gly^ in sgRNA synthesis. This system proved to be highly effective when *pks* and *xyr1* were used as two model target genes. The CRISPR–Cas9 system provides a technical platform for further study of the regulation mechanism of cellulase expression in *H. insolens*, which enables us to study the function of other transcriptional regulators and cellulase genes. It is expected to aid in promoting studies on regulation mechanisms of cellulase expression and engineering industrial strains with improved cellulase-producing ability.

## Material and methods

### Strains and culture media

The *H.  insolens* Y1  (CGMCC 4573) and its engineered strains were cultured on potato dextrose agar (PDA) plates at 42 °C for 5 days for conidiation. The yeast extract–peptone–dextrose medium (YPD) was used for mycelia growth at 42 °C. For cellulase production in flask fermentation, *H. insolens* were cultured at 42 °C for 6 days in a modified Melin-Norkrans medium (MMN) (containing 1 g/L tryptone, 20 g/L yeast extract, 0.6 g/L MgSO_4_·7H_2_O and 20 g/L Avicel). For observation of colony phenotypes, 2 × 10^5^ spores each of the *H. insolens* wild-type strain and its mutants were spotted and cultured on MMN, PDA, or YPD plates for 3 days at 42 °C. The *Escherichia coli* Top 10 (GenStar, Beijing, China) was grown at 37 °C for plasmid propagation in a Luria–Bertani (LB) broth supplemented with 100 μg/mL of ampicillin when necessary.

### Plasmid construction

The expressing cassette P*tef1*-NLS-*cas9*-NLS-T*trpC* containing the *tef1* promoter, a codon-optimized *cas9* gene with two nuclear localization signals (NLS), and the *trpC* terminator was synthesized according to the sequence of Cas9-expressing cassette used in *M. thermophila* as described earlier [17] for expression in *H. insolens*.

Two different sgRNA-expressing cassettes were constructed in this study. First, the *A. niger* 5S rRNA along with its 338-bp upstream promoter sequence, an HDV ribozyme, and a sgRNA scaffold were all amplified from the plasmid psgRNA4.0 as described earlier [25]. The second construct was constructed by replacing the HDV ribozyme with the tRNA^Gly^ fragment (sequence listed in Additional file 1: Table S2) from *H. insolens* (Fig. 1), which was predicted out of the genome by using the online GtRNAdb server (http://gtrnadb.ucsc.edu/) and amplified from the genomic DNA of *H. insolens* using a pair of primers tRNA^Gly^S/tRNA^Gly^A (Additional file 1: Table S1)*.* The sgRNA targeting sites for *pks* (GenBank accession number: MT875153) and *xyr1* (MT720880) genes in *H. insolens* were analyzed using the sgRNACas9 tool [65]. For *pks*, three different sgRNAs were analyzed to verify the stability of gene editing efficiency. The 23-nt protospacer sequences were 5′-GGCCTCCTCCACATTTGAGCAGG-3′ (*pks1*, the underline letter represents the PAM sequence), 5′-TCCCCGAAGAGGAGAAATGCAGG-3′ (*pks2*), and 5′-GAGGAGAAATGCAGGCTGCTCGG-3′ (*pks3*), respectively. For *xyr1*, the 23-nt protospacer sequence was 5′-CCCTTATGGTCCTGCTGCCAGGG-3′. The sgRNA fragments were in vitro annealed using synthesized primers shown in Additional file 1: Table S1 and individually cloned into a pBlunt-simple cloning vector, which yielded the plasmids pBS-5SHDVsgRNA-*pks1*, pBS-5SHDVsgRNA-*xyr1*, pBS-5StRNAsgRNA-*pks1*, and pBS-5StRNAsgRNA-*xyr1*, respectively. The plasmids pBS-5SsgRNA-*pks1*-HDV, pBS-5SsgRNA-*pks1*, pBS-tRNAsgRNA-*pks1*, pBS-5SHDVsgRNA-*pks2*, pBS-5SHDVsgRNA-*pks3*, pBS-5StRNAsgRNA-*pks2*, pBS-5StRNAsgRNA-*pks3* were constructed using the same method.

The 600-bp fragments flanking the targeting sites in donor DNAs (Fig. 1) were amplified by PCR from the *H. insolens* genomic DNA with primers shown in Table S1. The selection marker PT*gpd*-*hyg* containing the hygromycin resistance gene was amplified from the plasmid pAg1-*hyg* [8]. The 5′-flanking fragment, PT*gpd*-*hyg*, 3′-flanking fragment, and the *Not*I/*Xho*I-digested pBluescript KS were assembled using a Vazyme ClonExpress Ultra One Step Cloning Kit (Vazyme, Nanjing, China) to generate the plasmids pPT*gpd*-*hyg*-*Δpks* and pPT*gpd*-*hyg*-*Δxyr1* containing the donor DNA fragments “donor-*pks*” (for *pks* deletion) and “donor-*xyr1*” (for *xyr1* deletion), respectively.

### Protoplast transformation of *H. insolens*

Both the *H. insolens* wild-type Y1 and the Y1/*Δku70* strain were used as hosts in this study. Protoplast transformation of *H. insolens* was carried out as described earlier [66] with slight modifications. *H. insolens* strains were cultured on PDA medium at 42 °C for 3 days, and 10^7^ spores were harvested and transferred to the YPD medium for a continued culture of 10 h. Lysing enzymes (5 mg/mL) from *Trichoderma harzianum* (Sigma, L-1412) was used for releasing protoplasts from mycelia. The PDA medium supplemented with 50 µg/ml of Hygromycin B and 0.44 M of sucrose was used to screen for successful transformants.

For *pks* disruption, a total of 20–30 μg DNA including the PCR products of P*tef1*-NLS-*cas9*-NLS-T*trpC* (10 μg, amplified with the primer pair Cas9S/Cas9A, Additional file 1: Table S1), 5SHDVsgRNA-*pks1* or 5StRNAsgRNA-*pks1* (10 μg, both amplified with primers 5SHPS/5SSA, Additional file 1: Table S1), with or without the donor for *pks1* (10 μg, amplified with primers *pks*LS/*pks*RA, Additional file 1: Table S1) were mixed and added to the protoplasts of WT or Y1/*Δku70*. Transformants were screened on the PDA/hygromycin B plates and verified for DNA integration via PCR with primers shown in Additional file 1: Table S1. For *xyr1* disruption, a total of 30 μg DNA fragments including the PCR products of P*tef1*-NLS-*cas9*-NLS-T*trpC* (10 μg, obtained with the primer pair Cas9S/Cas9A), 5SHDVsgRNA-*xyr1* or 5StRNAsgRNA-*xyr1* (10 μg each, both amplified with primers 5SHPS/5SSA), and donor-*xyr1* (10 μg, amplified with the primer pair *xyr1*LS/*xyr1*RA) were similarly mixed and co-transformed into Y1/*Δku70*. The same procedure was used for transformations with other combinations of the DNA fragments (Table 1).

For simultaneous disruption of two genes, a total of 50 μg DNA including the PCR products of P*tef1*-NLS-*cas9*-NLS-T*trpC*, 5StRNAsgRNA-*pks1*, 5StRNAsgRNA-*xyr1*, donor (for *pks1*), and donor (for *xyr1*) (10 μg for each) were co-transformed into Y1/*Δku70*.

### Assay of enzyme activity and SDS-PAGE

The mycelia of *H. insolens* Y1 and mutant strains were individually cultured in YPD and then transferred to MMN for cellulase induction [9]. The cellulase activities including the overall cellulase activity (FPase, using filter paper as the substrate), the endoglucanase (or carboxymethyl cellulose activity, using carboxylmethyl cellulose as the substrate), cellobiohydralase (using *p*-nitrophenyl-β-cellobioside as the substrate), β-glucosidase (using *p*-nitrophenyl-β-glucopyranoside as the substrate), and xylanase activity (using birchwood xylan as the substrate) in the culture supernatant were determined according to the methods described before [67]. The extracellular proteins were resolved by SDS-PAGE on 12% (w/v) polyacrylamide gels. Proteins were visualized by staining with Coomassie Brilliant Blue G-250.

## Supplementary Information

**Additional file 1**: **Table S1:** Primers used in this study; **Table S2:** The DNA sequence of the tRNA^Gly^ from *H. insolens*; **Figure S1:** Multiple amino acid sequence alignment of Pks from *H. insolens* with four Pks homologs; **Figure S2:** Multiple amino acid sequence alignment of Xyr1 from *H. insolens* with four Xyr1 homologs.

## Data Availability

All data generated or analyzed during this study are included in this published article and its Additional files.
