# Supplementary material for: CRISPR/Cas9-mediated genome editing directed by a 5S rRNA–tRNAGly hybrid promoter in the thermophilic filamentous fungus Humicola insolens
Source: Biotechnol Biofuels. 2021 Oct 23;14:206. doi: 10.1186/s13068-021-02057-y (PMC8542335; doi:10.1186/s13068-021-02057-y)
Supplement: Supplementary file 1 — Additional file 1: Table S1: Primers used in this study; Table S2: The DNA sequence of the tRNAGly from H. insolens; Figure S1: Multiple amino acid sequence alignment of Pks from H. insolens with four Pks homologs; Figure S2: Multiple amino acid sequence alignment of Xyr1 from H. insolens with four Xyr1 homologs. [file 13068_2021_2057_MOESM1_ESM.doc]

**Additional file 1**

**Supplementary Tables**

**Table S1. Primers used in this study**

| **Primer** | **Sequence (5' to 3') ^a^** | **Note** |
| --- | --- | --- |
| Cas9S | GGCTCCCGCGACAGTATCTG | For PCR amplification of Cas9 expression cassette |
| Cas9A | GAAGAATAAGCGTGTCCCGA | For PCR amplification of Cas9 expression cassette |
| 5STHPS | GGTTGGAGATTCCAGACTCA | For construction of sgRNA expression cassette |
| 5SSA | AAAAAAGCACCGACTCGGTG | For construction of sgRNA expression cassette |
| PNHTS | GCGGCCGCCAGTGCTGCACA | For construction of Hyg expression cassette |
| PNHTA | CTCGAGACCAACCCAACCTC | For construction of Hyg expression cassette |
| tRNA^Gly^S | AACAAAGCGCCAGTGGTTTAGTGGTAAAATTCATCGTTGCCATCGATGAGCCCCGC | For synthesis of tRNA^Gly^ gene fragments |
| tRNA^Gly^A | TGCGCCAGCCGCGAATCGAACGCGGGGCTCATCGATGGCAACGATGAAT | For synthesis of tRNA^Gly^ gene fragments |
| 5SHP*xyr1*A | TGGCAGCAGGACCATAAGGGGGTGTTTCGTCCTTTCTCTG | For construction of *xyr1*-5SHDV-sgRNA expression cassette |
| 5SS*xyr1*S | CCCTTATGGTCCTGCTGCCAGTTTTAGAGCTAGAAATAGC | For construction of *xyr1*-5SHDV-sgRNA expression cassette |
| 5STP*xyr1*A | TACCACTAAACCACTGACGCTGCGCCAGCCGCGAATCGAA | For construction of *xyr1*-5StRNAsgRNA expression cassette |
| 5STS*xyr1*S | TTCGATTCGCGGCTGACGCAGGACGAAACCGAGGATGAGC | For construction of *xyr1*-5StRNAsgRNA expression cassette |
| *xyr1*LS | TAGGGCGAATTGGAGCTCCACCGCGGTGGCGTTATGCCCTGTCACTGACC | For PCR amplification of *xyr1*-armL |
| *xyr1*LA | CTGTATGTGCAGCACTGGCGGCCGCTGCTGCCGATGTTTTGGTTG | For PCR amplification of *xyr1*-armL |
| *xyr1*RS | GGGTGGAGGTTGGGTTGGTCTCGAGGAATTAGCAGGGCTTGTGAT | For PCR amplification of *xyr1*-armR |
| *xyr1*RA | ACAAAAGCTGGGTACCGGGCCCCCCCTCGACATAGGGGTGAACGGCGGTC | For PCR amplification of *xyr1*-armR |
| *xyr1*YS | TAGGTCGAAGATCCCGTCTC | For PCR verification of *xyr1* mutants |
| *xyr1*YA | GCTGGTAGGCGGTCGTGTTC | For PCR verification of *xyr1* mutants |
| 5STTS | CGAATCCCTTCTGTTGTATGAACAAAGCGCCAGTGGTTTAGTGGTA | For construction of *pks1*-5StRNAsgRNA expression cassette |
| 5STP*pks1*A | GCTCAAATGTGGAGGAGGCCTGCGCCAGCCGCGAATCGAA | For construction of *pks1*-5StRNAsgRNA expression cassette |
| 5STS*pks1*S | GGCCTCCTCCACATTTGAGCGTTTTAGAGCTAGAAATAGC | For construction of *pks1*-5StRNAsgRNA expression cassette |
| *pks1*LS | TAGGGCGAATTGGAGCTCCACCGCGGTGGCGTAGGATGGATGGGCTATGC | For PCR amplification of *pks1*-armL |
| *pks1*LA | CTGTATGTGCAGCACTGGCGGCCGCATCTTGGATGCGGAAGCCCC | For PCR amplification of *pks1*-armL |
| *pks1*RS | GGGTGGAGGTTGGGTTGGTCTCGAGTCGGGGACCAGTCACTCGAT | For PCR amplification of *pks1*-armR |
| *pks1*RA | ACAAAAGCTGGGTACCGGGCCCCCCCTCGATTGGGCTTCATCTTGGTCGA | For PCR amplification of *pks1*-armR |
| *pks1*YS | TCTACCTAAACACGTCCGGG | For PCR verification of *pks* mutants |
| *pks1*YA | TCCACCTCGGGATCGTCCAA | For PCR verification of *pks* mutants |
| 5ST*pks*2A | GCATTTCTCCTCTTCGGGGATGCGCCAGCCGCGAATCGAA | For construction of *pks2*-5StRNAsgRNA expression cassette |
| 5STH*pks*2S | TCCCCGAAGAGGAGAAATGCGTTTTAGAGCTAGAAATAGC | For construction of *pks2*-5SHDVsgRNA and 5StRNAsgRNA expression cassette |
| 5ST*pks*3A | AGCAGCCTGCATTTCTCCTCTGCGCCAGCCGCGAATCGAA | For construction of *pk3*-5StRNAsgRNA expression cassette |
| 5STH*pks*3S | GAGGAGAAATGCAGGCTGCTGTTTTAGAGCTAGAAATAGC | For construction of *pks3*-5SHDVsgRNA and 5StRNAsgRNA expression cassette |
| 5SH*pks*2A | GCATTTCTCCTCTTCGGGGAGGTGTTTCGTCCTTTCTCTG | For construction of *pks2*-5SHDVsgRNA expression cassette |
| 5SH*pks*3A | AGCAGCCTGCATTTCTCCTCGGTGTTTCGTCCTTTCTCTG | For construction of *pks3*-5SHDVsgRNA expression cassette |
| 5S*pks*H1 | GAGGCCGGTGTTTCGTCCTTTCATACAACAGAAGGGATTCG | For construction of *pks1*-5SsgRNA-HDV expression cassette |
| 5S*pks*H2 | CGAATCCCTTCTGTTGTATGAAAGGACGAAACACCGGCCTC | For construction of *pks1*-5SsgRNA-HDV expression cassette |
| 5S*pks*H3 | AAGTGGCACCGAGTCGGTGCGGACAACGAAATCGGCCTCT | For construction of *pks1*-5SsgRNA-HDV expression cassette |
| 5S*pks*H4 | AGAGGCCGATTTCGTTGTCCGCACCGACTCGGTGCCACTT | For construction of *pks1*-5SsgRNA-HDV expression cassette |
| 5S*pks*H5 | AAAAAACTCTGCATTGGCCTGATCAAAC | For construction of *pks1*-5SsgRNA-HDV expression cassette |
| tRNAS | GCGTCAGTGGTTTAGTGGTA | For construction of *pks1*-tRNAsgRNA expression cassette |

**Table S2. The DNA sequence of the tRNA^Gly^ from *H. insolens***

| **Gene** | **Sequence (5' to 3')** |
| --- | --- |
| tRNA^Gly^ | GCGCCAGTGGTTTAGTGGTAAAATTCATCGTTGCCATCGATGAGCCCCGCGTTCGATTCGCGGCTGGCGCA |

**Supplementary Figures**

**Figure S1. Multiple amino acid sequence alignment of Pks from *H. insolens* with four Pks homologs.** PfmaE (UniProt accession number W3X7U2, [1]), AnWA (Q03149, [2]), AnAlbA (A2QUI2, [3]) and AfAlb1 (Q4WZA8, [4]) are homologous protein of Pks from *P. fici*, *A. nidulans*, *A. niger* and *A. fumigatus*, respectively.

**Figure S2. Multiple amino acid sequence alignment of Xyr1 from *H. insolens* with four Xyr1 homologs.** MtXyr1 (UniProt accession number, G2QL41, [5]), NcXyr1 (Q7SAH8, [6]), TrXyr1 (G0RLE8, [7]) and AnXyr1 (Q5AVS0, [8]) are the Xyr1 homologous proteins from *M. thermophila*, *N. crassa*, *T. reesei* and *A. nidulans*, respectively.

**References**

1. Zhang P, Zhou S, Wang G, An Z, Liu X, Li K, Yin WB. Two transcription factors cooperatively regulate DHN melanin biosynthesis and development in *Pestalotiopsis fici*. Mol Microbiol. 2019; 112:649-666.

2. Fujii I, Watanabe A, Sankawa U, Ebizuka Y. Identification of Claisen cyclase domain in fungal polyketide synthase WA, a naphthopyrone synthase of *Aspergillus nidulans*. Chem Biol. 2001; 8:189-197.

3. Obermaier S, Müller M. Biaryl-forming enzymes from *Aspergilli* exhibit substrate-dependent stereoselectivity. Biochemistry. 2019; 58:2589-2593.

4. Jackson JC, Higgins LA, Lin X. Conidiation color mutants of *Aspergillus fumigatus* are highly pathogenic to the heterologous insect host *Galleria mellonella*. PLoS One. 2009; 4:e4224.

5. Berka RM, Grigoriev IV, Otillar R, Salamov A, Grimwood J, Reid I, Ishmael N, John T, Darmond C, Moisan MC, Henrissat B, Coutinho PM, Lombard V, Natvig DO, Lindquist E, Schmutz J, Lucas S, Harris P, Powlowski J, Bellemare A, Taylor D, Butler G, de Vries RP, Allijn IE, van den Brink J, Ushinsky S, Storms R, Powell AJ, Paulsen IT, Elbourne LD, Baker SE, Magnuson J, Laboissiere S, Clutterbuck AJ, Martinez D, Wogulis M, de Leon AL, Rey MW, Tsang A. Comparative genomic analysis of the thermophilic biomass-degrading fungi *Myceliophthora thermophila* and *Thielavia terrestris*. Nat Biotechnol. 2011; 29:922.

6. Galagan JE, Calvo SE, Borkovich KA, Selker EU, Read ND, Jaffe D, FitzHugh W, Ma LJ, Smirnov S, Purcell S, Rehman B, Elkins T, Engels R, Wang S, Nielsen CB, Butler J, Endrizzi M, Qui D, Ianakiev P, Bell-Pedersen D, Nelson MA, Werner-Washburne M, Selitrennikoff CP, Kinsey JA, Braun EL, Zelter A, Schulte U, Kothe GO, Jedd G, Mewes W, Staben C, Marcotte E, Greenberg D, Roy A, Foley K, Naylor J, Stange-Thomann N, Barrett R, Gnerre S, Kamal M, Kamvysselis M, Mauceli E, Bielke C, Rudd S, Frishman D, Krystofova S, Rasmussen C, Metzenberg RL, Perkins DD, Kroken S, Cogoni C, Macino G, Catcheside D, Li W, Pratt RJ, Osmani SA, DeSouza CP, Glass L, Orbach MJ, Berglund JA, Voelker R, Yarden O, Plamann M, Seiler S, Dunlap J, Radford A, Aramayo R, Natvig DO, Alex LA, Mannhaupt G, Ebbole DJ, Freitag M, Paulsen I, Sachs MS, Lander ES, Nusbaum C, Birren B. The genome sequence of the filamentous fungus *Neurospora crassa*. Nature. 2003; 422:859-868.

7. Martinez D, Berka RM, Henrissat B, Saloheimo M, Arvas M, Baker SE, Chapman J, Chertkov O, Coutinho PM, Cullen D, Danchin EG, Grigoriev IV, Harris P, Jackson M, Kubicek CP, Han CS, Ho I, Larrondo LF, de Leon AL, Magnuson JK, Merino S, Misra M, Nelson B, Putnam N, Robbertse B, Salamov AA, Schmoll M, Terry A, Thayer N, Westerholm-Parvinen A, Schoch CL, Yao J, Barabote R, Nelson MA, Detter C, Bruce D, Kuske CR, Xie G, Richardson P, Rokhsar DS, Lucas SM, Rubin EM, Dunn-Coleman N, Ward M, Brettin TS. Genome sequencing and analysis of the biomass-degrading fungus *Trichoderma reesei* (syn. *Hypocrea jecorina*). Nat Biotechnol. 2008; 26:553-560.

8. Tamayo EN, Villanueva A, Hasper AA, de Graaff LH, Ramón D, Orejas M. CreA mediates repression of the regulatory gene xlnR which controls the production of xylanolytic enzymes in *Aspergillus nidulans*. Fungal Genet Biol. 2008; 45:984-993.
